# Temporal trends and regional variations in hepatocellular carcinoma etiology: a multinational study across Asia

**DOI:** 10.1007/s12072-026-11037-z

**Published:** 2026-02-05

**Authors:** Yasuto Takeuchi, Ryosuke Tateishi, Shuntaro Obi, Motoyuki Otsuka, Hitoshi Mochizuki, Amarsanaa Jazag, Osamu Yokosuka, Sadahisa Ogasawara, Naoya Kanogawa, Erdenebayar Gonchig, Yuki Matsushita, Murat Kekilli, Gulden Bilican, Yoon Jun Kim, Moon Haeng Hur, Ming-Lung Yu, Chung-Feng Huang, Asahiro Morishita, Kyoko Oura, Atsumasa Komori, Yasuhide Motoyoshi, Lianda Siregar, Imelda Loho, Ming Yang, Shuichirou Okabe, Yoshiyuki Ueno, Tomohiro Katsumi, Takahisa Sato, Hirofumi Kogure, Ryota Masuzaki, Cosmas Rinaldi Lesmana, S. H. H. Nababan, Supot Nimanong, Yi-Hsiang Huang, Hiroaki Nagamatsu, Hideo Yoshida, Koji Uchino, Irsan Hasan, Taya Kitiyakara, Masatoshi Akamatsu, Makoto Okamoto, Mayuko Kondo, Makoto Moriyama, Wattana Sukeepaisarnjareon, Pisit Tangkijvanich, Kessarin Thanapirom, Kendal Yalcin, Hayriye Dilan Kızıl, Gupse Adali, Meral Akdogan, Dilara Turan Gokce, Yasemin Balaban, Hale Gokcan, Arif Mansur Coşar, Murat Harputluoglu, İbrahim Halil Bahçecioğlu, Alp Atasoy, Muhammet Cem Kockar, Hakan Dursun, Ahmet Tarik Eminler, Jose Sollano, Dong Jin Su, Teerha Piratvisuth, Jia-Horng Kao, Darrell Crawford, Jinlin Hou, Barjesh Chander Sharma, Diana Alcantara Payawal, Rino Alvani Gani, Tawesak Tanwandee, Jin Mo Yang, Han-Chieh Lin, Shuichiro Shiina, Ji-Dong Jia, George Lau, Necati Örmeci, A. Kadir Dokmeci, Lai Wei, Shiv Kumar Sarin, Masao Omata

**Affiliations:** 1 Okayama University School of Medicine, 2-5-1 Shikata-Cho, Kita-Ku, Okayama, 700-8558 Japan; 2 The University of Tokyo, 7-3-1, Hongo, Bunkyo-Ku, Tokyo, Japan; 3 Teikyo University Chiba Medical Center, Ichihara, Japan; 4 Yamanashi Central Hospital, kofu, Japan; 5 Happy Veritas Hospital, Ulan Bator, Mongolia; 6 Matsudo City General Hospital, Matsudo, Japan; 7 Chiba University School of Medicine, Chiba, Japan; 8 Gazi University, Ankara, Türkiye; 9 Seoul National University Hospital, Seoul, Republic of Korea; 10 Kaohsiung Medical University Hospital, Kaohsiung, Taiwan; 11 Kagawa University School of Medicine, Kagawa, Japan; 12 NHO Nagasaki Medical Center, Nagasaki, Japan; 13 Dharmais National Cancer Center, Jakarta, Indonesia; 14 Beijing Tsinghua Changgung Hospital, Beijing, China; 15 Yamagata University School of Medicine, Yamagata, Japan; 16 Nihon University School of Medicine, Tokyo, Japan; 17 Mochtar Riady Comprehensive Cancer Center Siloam Hospitals Semanggi, Jakarta, Indonesia; 18 Faculty of Medicine Siriraj Hospital, Mahidol University, Nakhon Pathom, Thailand; 19 Taipei Veterans General Hospital, Taipei, Taiwan; 20 Juntendo University School of Medicine, Tokyo, Japan; 21 Japanese Red Cross Medical Center, Tokyo, Japan; 22 Cipto Mangunkusumo General Hospital, Jakarta, Indonesia; 23 Faculty of Medicine Ramathibodi Hospital Mahidol University, Bangkok, Thailand; 24 JR Tokyo General Hospital, Tokyo, Japan; 25 Mitsui Memorial Hospital, Tokyo, Japan; 26 Khon Kaen University, Khon Kaen, Thailand; 27 Chulalongkorn University, Bangkok, Thailand; 28 King Chulalongkorn Memorial Hospital, Bangkok, Thailand; 29 Dicle University, Diyarbakır, Türkiye; 30 University of Health Sciences, Umraniye Training and Research Hospital, Istanbul, Türkiye; 31 Ankara Bilkent City Hospital, Ankara, Türkiye; 32 Hacettepe University, Ankara, Türkiye; 33 Ankara University, Ankara, Türkiye; 34 Karadeniz Technical University, Trabzon, Türkiye; 35 Inonu University, Malatya, Türkiye; 36 Fırat University, Elazığ, Türkiye; 37 Bahcesehir University Goztepe Medicalpark Hospital, Istanbul, Türkiye; 38 Suleyman Demirel University, Isparta, Türkiye; 39 Ataturk University, Erzurum, Türkiye; 40 Sakarya University, Adapazarı, Türkiye; 41 University of Santo Tomas Hospital, Manila, Philippines; 42 Daehang Hospital, Seoul, Korea; 43 Prince of Songkla University, Hat Yai, Thailand; 44 National Taiwan University Hospital, Taipei, Taiwan; 45 The University of Queensland, Brisbane, Australia; 46 Nanfang Hospital, Southern Medical University, Guangzhou, China; 47 Govind Ballabh Pant Institute of Postgraduate Medical Education and Research, New Delhi, India; 48 Fatima University Medical Center, Valenzuela, Philippines; 49 Yonsei University Health System, Seoul, Republic of Korea; 50 Beijing Friendship Hospital, Capital Medical University, Beijing, China; 51 Humanity & Health Medical Group, Hong Kong, China; 52 İstanbul Health and Technology University, Istanbul, Türkiye; 53 Tsinghua University, Beijing, China; 54 Institute of Liver and Biliary Sciences, New Delhi, India

**Keywords:** Hepatocellular carcinoma, Epidemiology, Temporal trend, Asia

## Abstract

**Background:**

Hepatocellular carcinoma (HCC) remains a major health burden in Asia. Advances in antiviral therapies are reshaping the etiological landscape of HCC. This study evaluated temporal shifts in HCC etiology across Asian countries and their clinical implications.

**Methods:**

This multinational study analyzed 6,261 newly diagnosed HCC patients registered in the APASL Hepatology/Oncology Consortium (A-HOC) from 19 centers across seven Asian countries and regions between 2013 and 2023. Data on demographics, tumor characteristics, etiology, and treatment patterns were collected. Etiologies included hepatitis B virus (HBV), hepatitis C virus (HCV), alcoholic liver disease (ALD), metabolic dysfunction-associated fatty liver disease (MAFLD), MAFLD plus excess alcoholic intake (MAFLD + eAL), autoimmune liver disease, cryptogenic, and others. Temporal trends and regional variations were assessed.

**Results:**

In many countries, HBV remained predominant (43.3%–69.5%) and relatively stable throughout the period, while HCV showed only modest reductions. In Japan, HCV was the leading cause of HCC (33.1%), with a significant decline over time, accompanied by a rise in MAFLD-related HCC. ALD-related HCC increased in South Korea, and MAFLD-related HCC rose in Turkey. Tumor size and stage at diagnosis varied by etiology and region, affecting treatment strategies. Early-stage diagnosis was more frequent in Japan and Taiwan, whereas advanced-stage HCC was common in China and Indonesia.

**Conclusions:**

Distinct regional patterns and temporal changes in HCC etiology across Asia highlight the need for tailored prevention and surveillance measures. The growing burden of MAFLD-related HCC emphasizes its emerging role in liver cancer development, particularly in regions with declining viral hepatitis.

**Supplementary Information:**

The online version contains supplementary material available at 10.1007/s12072-026-11037-z.

## Introduction

Hepatocellular carcinoma (HCC) is one of the most prevalent cancers worldwide. According to the World Health Organization (WHO), 865,835 new cases of HCC were identified globally in 2022, making it the third leading cause of cancer-related mortality [1]. In Asia, 607,266 new cases of HCC were reported in 2022, accounting for 70.1% of all newly diagnosed cases worldwide. HCC remains a critical public health concern in Asia, with 530,835 liver cancer-related deaths recorded in the same year [1].

HCC is a complex and aggressive malignancy with multiple etiological factors. [2] In recent years, the widespread implementation of universal hepatitis B virus (HBV) vaccination, the use of nucleoside/nucleotide analogs, and the development of direct-acting antiviral agents (DAAs) for hepatitis C virus (HCV) have facilitated the prevention, control, and potential elimination of viral hepatitis infections. As a result, the etiological landscape of HCC is shifting globally, including in Asia, due to an increasing prevalence of fatty liver disease associated with lifestyle changes [3]. However, as the etiology of HCC varies by region, region-specific approaches are essential for its prevention and surveillance [4]. Nevertheless, to date, no study has systematically investigated temporal changes in the etiological factors of hepatocellular carcinoma (HCC) in the Asian region.

The objective of this study is to investigate the temporal changes in the etiology of HCC across different regions in Asia.

## Patients and methods

### Study design

This study is part of the APASL Hepatology/Oncology Consortium (A-HOC), a multinational registry involving multiple medical institutions across Asia (Supplementary Table 1) that collects real-world data on patients with HCC; the overall study design, data structure, and IRB approval process have been previously reported.

### Patients

This observational study collected data from the “Survey on Current Status and Treatment of Hepatitis and Liver Cancer in the Asia–Pacific Region” involving 6,261 participants from January 1, 2013 to December 31, 2023. Patients aged 18 years or older who visited one of the 19 participating facilities and were newly diagnosed with HCC were included. The diagnosis followed the APASL clinical practice guidelines for HCC. [5] All treatment decisions and clinical management of patients were determined by each site and at the discretion of the treating physician.

### Data collection

Prior to data collection, information about the study was disclosed on the hospitals’ website, allowing patients the opportunity to refuse participation, in accordance with the ethical guidelines of each participating site. The data included clinical characteristics, tumor status, and initial treatment. The initial treatment refers to the therapy introduced at the time of initial HCC diagnosis. The etiologies of the background liver diseases were classified as follows: HBV, HCV, alcoholic liver disease (ALD), metabolic dysfunction-associated fatty liver disease (MAFLD), MAFLD + excess alcoholic intake (MAFLD + eAL), autoimmune liver disease (Autoimmune), others, and cryptogenic. Daily alcohol intake was estimated using a standardized report form detailing alcohol consumption and frequency. In this study, MAFLD was diagnosed using the definition established by the International Expert Panel [6]. And excess alcohol intake was defined as a daily alcohol consumption of ≥ 30 g and < 60 g for men, and ≥ 20 g and < 60 g for women. We collected the study data using REDCap. Missing values were defined as data recorded as blank, not applicable, or outliers. Outliers were defined as values rounded to the 1st and 99th percentile for each variable. Missing data were processed by listwise deletion. Variables with missing values accounted for less than 10% of the dataset, and the missingness was assumed to be missing completely at random. The data are not publicly available due to ethical restrictions and the protection of patient privacy.

### Statistical analyses

All eligible patients registered in the study were included in the analysis population. Results of analyses of baseline clinical characteristics and initial treatment were reported as aggregate data for each country. Data are expressed as medians with interquartile ranges (25th to 75th percentiles). Numbers and percentages were used for qualitative variables. Trends in etiological proportions were reported for each country. The Cochran–Armitage trend test was used to evaluate increasing or decreasing trends. The Wilcoxon rank-sum test was used to evaluate the difference in tumor size between the groups. All statistical analyses were performed using R software (ver. 4.4.0; R Development Core Team, Vienna, Austria). All the tests were two-sided, and *p* values < 0.05 were considered to indicate statistical significance.

## Results

### Patient demographics

A total of 6,261 newly diagnosed HCC patients were recruited from 19 institutions across seven countries in Asia. The number of patients enrolled each year is shown in supplementary Figure S1. This study included 4,049 patients from Japan, 338 from China, 247 from South Korea, 457 from Taiwan, 509 from Turkey, 247 from Indonesia, and 414 from Mongolia. The clinical characteristics of patients from each country are also presented in Table 1.

**Table 1:** Patient Characteristics

	All patients (*n* = 6261)	Japan (*n* = 4049)	China (*n* = 338)	South Korea (*n* = 247)	Taiwan (*n* = 457)	Turkey (*n* = 509)	Indonesia (*n* = 247)	Mongolia (*n* = 414)
Age, year	69 (60–76)	73 (66–79)	56 (48–63)	65 (57–71)	65 (58–71)	62 (55–68)	56 (47–66)	60 (54–66)
Male, Gender, n (%)^a^	4548 (72.6)	2946 (72.8)	280 (82.8)	200 (81.0)	332 (72.6)	400 (78.6)	183 (74.1)	207 (50.0)
BMI (kg/m^2^)	23.9 (21.6–26.5)	23.6 (21.4–26.3)	22.9 (21.0–25.0)	24.4 (22.4–26.6)	24.5 (22.4–27.3)	26.3 (23.5–29.8)	22.2 (20.0–24.9)	25.1 (22.5–26.6)
Diabetes, n (%)^b^	1545 (24.7)	1035 (25.6)	65 (19.3)	55 (22.3)	165 (36.1)	164 (32.2)	51 (20.9)	10 (2.4)
Child–Pugh class^c^								
A, n (%)	4483 (71.7)	3050 (75.3)	235 (69.5)	233 (94.3)	419 (91.7)	284 (55.9)	80 (32.9)	182 (44.2)
B, n (%)	1036 (16.6)	707 (17.5)	51 (15.1)	12 (4.9)	33 (7.2)	145 (28.5)	45 (18.5)	43 (10.4)
C, n (%)	236 (3.8)	123 (3)	12 (3.6)	2 (0.8)	5(1.1)	70 (13.8)	14 (5.8)	10 (2.4)
Maximal tumor size (cm)	3.4 (2.0–6.5)	3.0 (2.0–5.7)	5.8 (3.1–10.1)	2.8 (1.7–5.8)	3.1 (2.1–5.8)	4.9 (2.9–8.5)	9.7 (6.4–12.6)	3.4 (2.4–5.0)
Number of nodules^d^								
Single, n (%)	3247 (52.5)	2324 (57.8)	119 (37.0)	172 (69.6)	269 (58.9)	224 (44.4)	38 (16.2)	101 (25.4)
2–3, n (%)	1634 (26.4)	862 (21.4)	94 (29.2)	51 (20.7)	110 (24.1)	151 (29.9)	97 (41.5)	269 (67.8)
> 3, n (%)	1303 (21.0)	836 (20.8)	109 (33.9)	11 (9.7)	78 (17.1)	130 (25.8)	99 (42.3)	27 (6.8)
Vascular invasion, n (%)^e^	699 (11.4)	331 (8.3)	109 (32.3)	32 (13.0)	56 (12.3)	107 (21.1)	61 (25.6)	3 (0.8)
Extrahepatic metastasis, n (%) ^f^	590 (9.6)	344 (8.6)	61 (18.2)	9 (3.6)	20 (4.4)	75 (14.7)	38 (16.0)	43 (11.3)
AFP (ng/mL)	14 (4.7–199)	11 (4.2–104)	379 (24–4570)	11 (4.3–90)	15 (4.0–130)	26 (5.0–439)	727 (16–12,082)	63 (12–639)
BCLC stage^g^								
0, n (%)	721 (11.6)	592 (14.6)	3 (0.9)	51 (20.6)	47 (10.3)	23 (4.5)	0 (0)	5 (1.3)
A, n (%)	2225 (35.8)	1668 (41.2)	66 (19.6)	114 (46.2)	176 (38.5)	156 (30.8)	9 (3.8)	36 (9.4)
B, n (%)	1283 (20.6)	769 (19)	62 (18.4)	50 (20.2)	119 (26)	137 (27.1)	125 (52.5)	21 (5.5)
C, n (%)	1250 (20.1)	789 (19.5)	116 (34.4)	32 (13)	108 (23.6)	130 (25.7)	74 (31.1)	1 (0.3)
D, n (%)	219 (3.5)	141 (3.5)	3 (0.9)	0 (0)	7 (1.5)	47 (9.3)	20 (8.4)	1 (0.3)
Initial treatment^h^								
Surgery, n (%)	1192 (19.1)	723 (17.9)	83 (24.6)	45 (18.2)	145 (32.3)	41 (8.1)	11 (4.5)	141 (34.1)
Ablation, n (%)	1365 (21.8)	1059 (26.2)	25 (7.4)	50 (20.2)	103 (22.5)	77 (15.1)	14 (5.7)	37 (8.9)
Embolization, n (%)	1818 (29.0)	1153 (28.5)	90 (26.6)	134 (54.3)	134 (29.3)	114 (22.4)	61 (24.7)	132 (31.9)
Radiation, n (%)	114 (1.8)	98 (2.4)	4 (1.2)	1 (0.4)	7 (1.5)	2 (0.4)	2 (0.8)	0 (0)
Systemic therapy, n (%)	597 (9.5)	320 (7.9)	76 (22.5)	17 (6.9)	28 (6.1)	78 (15.3)	52 (21.1)	26 (6.3)
Hepatic arterial infusion, n (%)	153 (2.4)	114 (2.8)	32 (9.5)	0 (0)	0 (0)	7 (1.4)	0 (0)	0 (0)
Heavy Particle, n (%)	17 (0.3)	17 (0.4)	0 (0)	0 (0)	0 (0)	0 (0)	0 (0)	0 (0)
Transplantation, n (%)	64 (1.0)	8 (0.2)	7 (2.1)	0 (0)	1 (0.2)	4 (9.4)	0 (0)	0 (0)
No treatment, n (%)	932 (14.9)	557 (13.8)	21 (6.2)	0 (0)	27 (5.9)	142 (27.9)	107 (43.3)	78 (18.8)

Data are expressed as the median (25th–75th percentiles) or number (percentages)

AFP alpha-fetoprotein, BCLC Barcelona Clinic Liver Cancer, BMI body mass index. Data were missing for a0, b13, c7, d0, e108, f108 g45 and h0 patients in the cohort, respectively

Among the enrolled patients, those from Japan exhibited the highest median age at 73 years. The proportion of male patients was generally high across countries, exceeding 70%. Turkey recorded the highest median body mass index at 26.3 kg/m^2^. The prevalence of diabetes varied substantially, with Taiwan (36.1%) and Turkey (32.2%) having relatively high rates.

Liver function status, as indicated by Child–Pugh class A, was best preserved in South Korea (94.3%) and Taiwan (91.7%). Patients from Indonesia presented with the largest median tumor size (9.7 cm), and a high proportion had advanced-stage disease, with BCLC-B and C accounting for over 80% of cases. Conversely, patients from Japan and South Korea tended to have smaller tumors (median size 3.0 cm and 2.8 cm, respectively) and were more frequently diagnosed at early stages (BCLC-0 or A). The median AFP level was notably low in Japan (11 ng/mL).

Treatment patterns varied by region. Embolization therapy was the most common initial treatment in several countries, particularly South Korea (54.3%). Surgical resection was frequently selected in Taiwan (32.3%) and Mongolia (34.1%). Notably, a significant proportion of patients in Indonesia (43.3%) received no initial treatment.

### Etiology of HCC in Asia

**Fig. 1:**
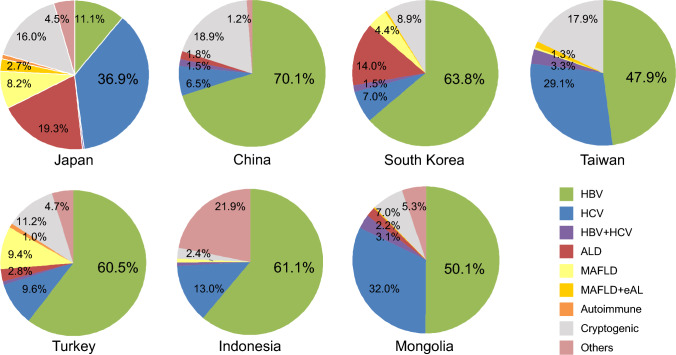
Etiology in patients with HCC in Asian countries from 2013 to 2023. In Japan, HCV accounted for the largest proportion of cases, whereas in other countries, HBV was the predominant etiology. The proportion of metabolic-related liver disease was higher in Japan, South Korea, and Turkey. Alcohol-related liver disease accounted for a consistent proportion of cases in both Japan and South Korea

The distribution of the etiology in Asia was primarily attributed to HBV (Fig. 1). When analyzed by country, HCV was the most common etiology in Japan (33.1%). MAFLD was 7.5% and MAFLD + eAL was 12.1%. In other countries, HBV was the predominant cause. The proportion of ALD was slightly higher in South Korea than in other countries, while MAFLD was more prevalent in Turkey. In contrast, the proportions of ALD and MAFLD were lower in Taiwan and Mongolia compared to other countries. These findings highlight significant differences in the etiology of HCC between Japan and other Asian countries.

Among 6,192 patients with available etiology data, 59.7% had viral hepatitis (HBV, HCV, or both), 13.8% had alcohol-related disease (ALD), 6.4% had metabolic dysfunction-associated fatty liver disease (MAFLD / MAFLD + eAL), and 20.2% had other etiologies (autoimmune, cryptogenic, or others). Patient characteristics significantly differed among these etiologic groups (all p < 0.01). Patients with viral etiology were younger (median 66 years) compared with ALD (71 years), MAFLD (73 years), and others (73 years). The proportion of male patients was highest in ALD (95.6%) and lowest in MAFLD (54.5%). MAFLD patients had the highest BMI (median 26.3 kg/m^2^) and the highest prevalence of diabetes (64.1%). Child–Pugh A predominated across all groups (67–78%), but Child–Pugh B/C were more frequent in ALD and others. Tumor size was largest in the “others” group (median 4.3 cm), and vascular invasion or extrahepatic spread were more common in viral cases. AFP levels were highest in viral and “others” etiologies. Initial treatment patterns also varied significantly (p < 0.01): surgical resection and ablation were more frequent in viral and MAFLD groups, whereas embolization was the most common first-line therapy in all groups (Supplementary Table S2).

### Regional and temporal trends in the etiology of HCC

**Fig. 2:**
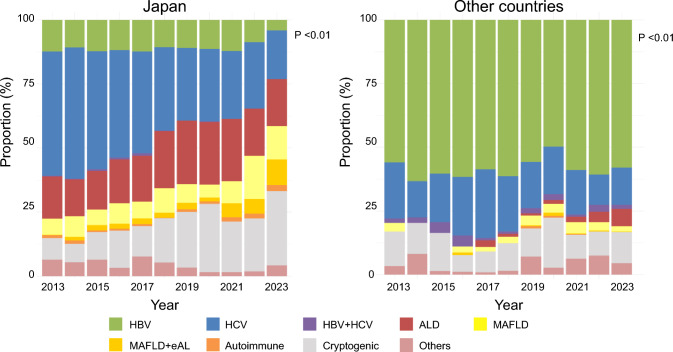
Trend in background liver diseases among patients with HCC in Japan and other countries. In Japan, the proportion of viral hepatitis, particularly HCV, showed a significant decrease (*P* < 0.01, Cochran–Armitage test). In contrast, while a decrease in HCV was observed in other countries (*P* < 0.01, Cochran–Armitage test), the proportion of HBV remained persistently high, resulting in no significant overall reduction in viral hepatitis

Annual trends in etiology reveal a significant decline in HCV and a gradual decrease in HBV in Japan, resulting in a statistically significant drop in viral hepatitis (*p* < 0.01, Cochran–Armitage test). Outside Japan, HCV also declined significantly (*p* < 0.01), while HBV remained consistently high. Overall, the proportion of viral hepatitis has not changed substantially. Outside Japan, ALD is rising but remains low. Japan shows increasing trends in MAFLD and MAFLD + eAL, whereas outside Japan, these proportions peaked in 2016 and then stabilized (Fig. 2).

When examining countries outside Japan individually, HBV remained high without significant decline, particularly in China. HCV gradually declined in China and Mongolia, but showed little change elsewhere. In South Korea, ALD increased, and both MAFLD and MAFLD + eAL showed gradual rises in South Korea and Turkey. In Taiwan and Indonesia, ALD remained low; although MAFLD + eAL temporarily increased, it later declined (Supplementary Figure S2).

### Chronological changes in BCLC stage

**Fig. 3:**
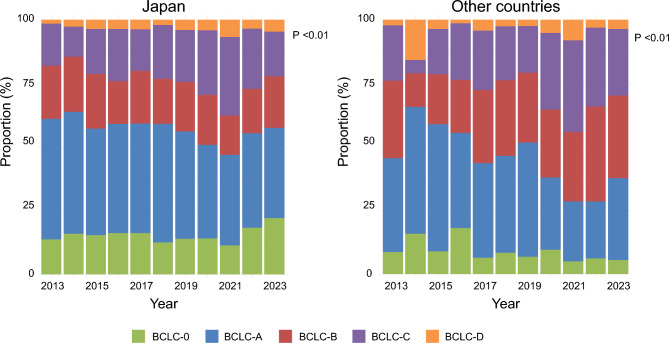
Trend of BCLC stage in Japan and other countries. In Japan, the proportion of early-stage HCC remained relatively stable, whereas in other countries, it showed a decreasing trend, accompanied by an increase in the proportion of BCLC-B. The proportion of BCLC-C demonstrated an increasing trend in both Japan and other countries (*P* < 0.01, Cochran–Armitage test)

Annual trends in BCLC stage were examined between Japan and countries outside Japan. In Japan, the overall proportion of early-stage HCC remained stable; however, a more detailed analysis revealed a gradual decrease in the proportion of BCLC-A and an increase in BCLC-0. While the proportion of BCLC-B showed a slight decline, BCLC-C, which represents a more advanced stage, demonstrated an increasing trend (p < 0.01, Cochran–Armitage test). In contrast, countries outside Japan exhibited different characteristics: the proportions of early-stage HCC (BCLC-0 and BCLC-A) decreased, whereas an increasing trend was observed not only in BCLC-C (*p* < 0.01, Cochran–Armitage test) but also in BCLC-B, which represents the intermediate stage (Fig. 3).

### Changes in initial treatment methods

The initial treatment approaches for HCC were studied. In Japan, the proportion of early-stage HCC remained relatively constant, resulting in minimal changes in the use of curative treatments such as surgical resection and ablation. Conversely, the rising proportion of advanced-stage HCC led to an increase in the use of systemic therapy (*p* < 0.01, Cochran–Armitage test). The percentage of patients receiving TACE gradually decreased (Supplementary Figure S3).

In China, resection and ablation decreased while HAIC and systemic therapy increased. South Korea saw a decline in resection and ablation but an increase in TACE and systemic therapy. Mongolia showed similar trends. Taiwan maintained high ablation rates, with decreased resection and increased systemic therapy. Turkey and Indonesia experienced declining resection and ablation, stable TACE, and rising systemic therapy. Both countries had higher supportive care rates than others (Supplementary Fig. S4).

### Tumor size characteristics by etiology across countries

**Fig. 4:**
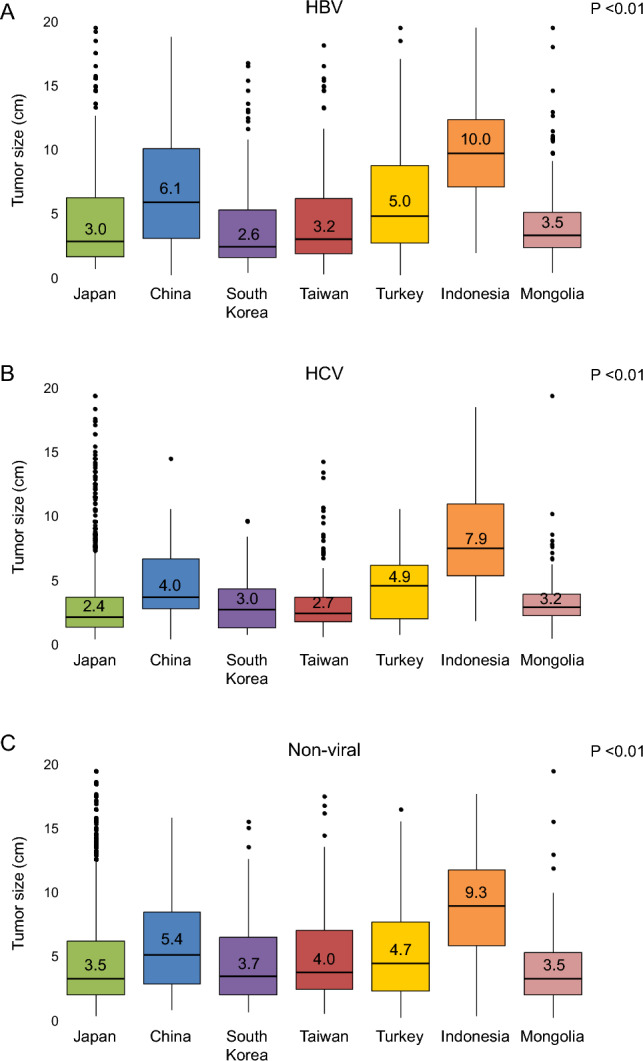
Tumor size by etiology and country. In many countries, HCC associated with HCV tended to present with smaller tumor sizes, whereas HCC arising from non-viral liver diseases tended to have larger tumor sizes. However, these patterns varied by country. Notably, China, Turkey, and Indonesia were characterized by generally larger tumor sizes compared to other countries

In all countries except Turkey, the median tumor size at initial diagnosis of HCC was the smallest among patients with HCV. This was a notable finding. In Turkey, the smallest median tumor size was observed in patients with non-viral liver disease. In Japan and South Korea, the second smallest tumor size was seen in patients with HBV, while the largest was observed in those with non-viral liver disease. In contrast, in China, Turkey, and Indonesia, patients with non-viral liver disease had the largest median tumor sizes. In Mongolia, there was no notable difference in tumor size between HBV and non-viral cases. Additionally, China, Turkey, and Indonesia were characterized by a generally larger median tumor size at initial HCC diagnosis compared to other countries (*p* < 0.01, Wilcoxon rank-sum test) (Fig. 4).

## Discussion

This study reveals regional variations in the etiology and clinical characteristics of HCC across Asia. Consistent with previous reports, [3] viral hepatitis remains the leading cause of HCC, with HCV predominant in Japan and HBV in other Asian countries. In Japan, virus-related HCC has markedly declined, largely due to the widespread use of DAAs, while such changes were not observed elsewhere. Regarding lifestyle-related causes, MAFLD-related HCC is rising in Japan and Turkey, while ALD is more common in South Korea. These findings emphasize the need for region-specific strategies for HCC prevention and management.

Several recent reports from a global or regional perspective have described temporal changes in the epidemiology of hepatocellular carcinoma and the growing contribution of metabolic and alcohol-related liver disease to HCC burden. [7–10] In contrast, the present study focuses specifically on inter-country differences within Asia, using harmonized, multicenter data from the A-HOC network. This approach provides a more detailed and regionally relevant comparison of patient profiles and management patterns across diverse healthcare systems in Asia, thereby highlighting the novelty of the current analysis.

In 2016, the World Health Organization (WHO) launched the Global Health Sector Strategy (GHSS), setting a goal to eliminate HCV as a public health threat by 2030 [11]. This initiative aims for a 90% reduction in new infections and a 65% reduction in HCV-related mortality compared to 2015 levels. Achieving these targets requires a significant expansion of screening and improved access to pan-genotypic DAAs. While countries like Japan have demonstrated substantial progress—with over a 50% reduction in HCV-related mortality [12]—many high-burden countries continue to lag behind [13]. Strengthening healthcare infrastructure, implementing targeted screening programs, and removing stigma and financial barriers are essential interventions to accelerate HCV elimination efforts across Asia.

GHSS also sets ambitious targets to eliminate hepatitis B as a public health threat by 2030: 90% reduction in new chronic infections, 65% reduction in HBV-related deaths, 90% birth-dose vaccination coverage, 90% diagnosis rate, and 80% treatment coverage for eligible individuals. Although some studies reported declining trends in HBV-related HCC incidence due to the widespread adoption of HBV vaccination [14], this finding may not fully represent the overall trend in Asia, as the study was limited to specific countries. Furthermore, individuals born after the introduction of HBV vaccination will be under 40 years of age by 2023, which is considerably younger than the typical age of HBV-related HCC onset. Our study revealed that the proportion of HCC related to HBV remains high in Asian countries, likely due to the low rate of antiviral treatment for HBV, another key preventive measure for HCC development [15]. Inadequate interventions against HBV, including insufficient HBV vaccination coverage and lack of disease awareness,[16] delayed implementation of screening, and disparities in access to treatment, are challenges that must be overcome to reduce HBV-related HCC.

This study emphasizes the expanding role of lifestyle-related liver diseases—particularly MAFLD, ALD, and their overlap—as etiological factors for HCC in Asia. [17] In our cohort, a significant increase in MAFLD-associated HCC was observed in both Japan and Turkey. Although HCC incidence has decreased in Japan [18] and increased in Turkey [19], both countries show a parallel rise in MAFLD-related HCC. [20, 21] In contrast, MAFLD-associated HCC remains relatively uncommon in other Asian countries, likely due to shorter exposure to metabolic risk factors and the persistently high prevalence of viral hepatitis. However, the increasing prevalence of obesity, type 2 diabetes, and metabolic syndrome across Asia is expected to drive a future rise in MAFLD-related HCC. [22] Given the estimated 30- to 40-year lag between increases in liver disease and HCC incidence [23], other Asian countries may soon face a similar rise. Finally, differences in national healthcare systems and the tertiary-care setting of participating institutions may have limited our ability to accurately capture the true prevalence of MAFLD-associated HCC.

ALD-related HCC remained largely stable, except for a slight increase in South Korea, consistent with increased alcohol consumption in that country [24]. While ALD prevalence is generally reported to be increasing across Asia, substantial regional variations have been noted [25]. For example, increasing trends have been observed in Japan, China, and Turkey, [26–28] whereas no clear trend has been seen in other countries. Although ALD is an established risk factor for HCC [29], its impact is often underestimated due to difficulties in quantifying alcohol intake and social stigma. These observed differences in ALD-related HCC may reflect genuine variations in exposure or differences in reporting practices across countries.

An important observation is the increasing prevalence of MAFLD + eAL, particularly in Japan. This group, now referred to as MetALD [30], is characterized by metabolic abnormalities and moderate alcohol consumption. Previously excluded from traditional etiological categories, this group is now recognized as being at high risk for both liver fibrosis and hepatocellular carcinoma. [31] The increasing incidence of HCC associated with MAFLD, ALD, and their overlap suggests that previously “cryptogenic” cases may reflect unrecognized lifestyle-related etiologies. Expanding HCC screening to incorporate metabolic history and alcohol use, along with implementing risk-based, individualized surveillance, is recommended [32].

This study identified differences in initial treatment trends across countries. As treatment decisions depend on liver function, tumor stage, and overall patient condition, direct comparisons are challenging. Notably, tumor size at diagnosis varied by country. Prior studies have also reported prognostic differences in HCC within Asia, [33] suggesting that tumor status at diagnosis may influence outcomes. The recent reduction in the proportion of patients receiving curative therapies such as resection or ablation observed in some countries may be explained by an increasing proportion of patients diagnosed at intermediate or advanced stages (BCLC-B and BCLC-C). This shift likely reflects changes in the underlying patient population, with a growing prevalence of non-viral etiologies and more advanced disease at presentation. While global societies provide generally consistent surveillance guidelines, [34] regional disparities in their implementation may contribute to variation in tumor progression at diagnosis. Although all participating centers follow the APASL recommendations for HCC surveillance, information on individual adherence or implementation status of surveillance programs was not available in this registry. Future updates of the A-HOC database are planned to include these data to allow more detailed analyses of their impact on tumor stage at diagnosis.

This study has several limitations. (1) The database used in this study was compiled from HCC data maintained by each participating facility. As the timing of database creation varied across facilities, the distribution of etiology in earlier data may be less consistent and therefore less reliable. (2) Because the database format differs between facilities, missing values were present for some survey items. (3) This study does not encompass all Asian countries. The analysis included data from seven Asian countries (Japan, China, Korea, Taiwan, Turkey, Indonesia, and Mongolia), which represent geographically and epidemiologically diverse regions across East, Southeast, and Western Asia. Therefore, although the findings may not be fully generalizable to the entire Asian continent, they are likely to capture major regional differences in HCC etiology and clinical characteristics. Despite these limitations, the large sample size and multicenter design enhance the generalizability of the findings. The completeness and stability of data collection varied among countries, which likely reflects differences in the comprehensiveness and maturity of institutional databases over time. In particular, Japan and Taiwan had more continuous and extensive data acquisition, whereas some participating countries showed temporal fluctuations in the registration of specific etiologies, such as MAFLD or ALD. These variations may have influenced the apparent year-to-year changes observed in certain countries, and should be interpreted with caution.

This study aimed to elucidate the evolving epidemiology of HCC in Asia and to underscore the importance of tailored prevention, diagnosis, and treatment strategies in each country. From a clinical and public health perspective, these findings underscore the need to strengthen surveillance and prevention strategies tailored to the evolving epidemiology of HCC in Asia. The increasing proportion of patients diagnosed at intermediate or advanced stages highlights gaps in current surveillance systems, particularly for non-viral etiologies such as MAFLD and alcohol-related liver disease. Expanding risk-based screening programs and integrating metabolic and lifestyle factors into surveillance criteria could improve early detection. In addition, public health initiatives targeting obesity, diabetes, and alcohol consumption will be essential to mitigate the future HCC burden in Asian populations. Further research is warranted to evaluate the impact of metabolic risk factors and the long-term effects of antiviral interventions.

## Supplementary Information

Below is the link to the electronic supplementary material.

Supplementary file1 (DOCX 24 kb)

## Abbreviations

AFP: Alpha-fetoprotein
ALD: Alcoholic liver disease
BCLC classification: Barcelona Clinical Liver Cancer classification
CI: Confidence interval
C–P: Child–Pugh
DAAs: Direct-acting antiviral agents
HAIC: Hepatic arterial infusion chemotherapy
HBV: Hepatitis B virus
HCC: Hepatocellular carcinoma
HCV: Hepatitis C virus
MAFLD: Metabolic dysfunction-associated fatty liver disease
TACE: Transarterial chemoembolization
