# Supplementary material for: Temporal trends and regional variations in hepatocellular carcinoma etiology: a multinational study across Asia
Source: Hepatol Int. 2026 Feb 5;20(2):373–83. doi: 10.1007/s12072-026-11037-z (PMC13121539; doi:10.1007/s12072-026-11037-z)
Supplement: Supplementary file 1 — Supplementary file1 (DOCX 24 kb) [file 12072_2026_11037_MOESM1_ESM.docx]

# SUPPLEMENTARY FIGURE LEGENDS

**Figure S1.** The trend in the number of patient registrations by fiscal year.

**Figure S2.** Trends in etiology by country. In Japan, the proportion of hepatitis virus-related cases has decreased over time, while the proportion of metabolic liver diseases has increased. In contrast, countries outside Japan still show high proportion of hepatitis virus-related cases, largely due to the continued prevalence of HBV. Notably, South Korea and Turkey have seen a recent rise in the proportion of alcoholic and metabolic liver diseases.

**Figure S3.** Annual trends in treatment modalities. In Japan, the use of TACE has been declining, while systemic therapy has been increasing. In countries outside Japan, systemic therapy is also on the rise; however, ablation therapy has decreased, and TACE shows an increasing trend.

**Figure S4.** Trends in initial treatment modalities for HCC across Asian countries. While systemic therapy increased in most countries over the study period, resection and ablation tended to decrease, particularly in South Korea, Turkey, and Indonesia. The proportion of patients receiving supportive care remained notably high in Indonesia and Turkey. Statistically significant trends were observed in several countries (P-values indicated).

# SUPPLEMENTARY TABLES

# Supplementary Table 1: A list of participating institutions

| Regions | Institutions |
| --- | --- |
| Japan | Yamanashi Central Hospital |
|  | The University of Tokyo Hospital |
|  | Okayama University Hospital |
|  | Chiba University Hospital |
|  | Nagasaki Medical Center |
|  | Kagawa University Hospital |
|  | Matsudo City Hospital |
|  | Teikyo University Hospital |
|  | Yamagata University Hospital |
|  | Juntendo University Hospital |
| China mainland | Beijing Tsinghua Changgung Hospital |
| Hong Kong SAR, China | Humanity & Health Medical Group |
| South Korea | Seoul National University Hospital |
| Taiwan | Kaohsiung Medical University Chung-Ho Memorial Hospital |
|  | Taipei Veterans General Hospital |
| Turkey | Gazi University |
| Indonesia | Dharmais National Cancer Center |
|  | Mochtar Riady Comprehensive Cancer Center Siloam Hospitals Semanggi |
| Mongolia | Happy Veritas Hospital |

# Supplementary Table 2: Patient background by etiology

|  | Viral  (n=3694) | ALD  (n=854) | MAFLD/  MAFLD+eAL  (n=396) | Others  (n=1248) | *P*-value |
| --- | --- | --- | --- | --- | --- |
| Age, year | 66 (58–74) | 71 (64–77) | 73 (66–79) | 73 (65–79) | < 0.01 |
| Male, Gender, n (%) ^a^ | 2600 (70.4) | 816 (95.6) | 216 (54.5) | 838 (67.1) | < 0.01 |
| BMI (kg/m^2^) | 23.6 (21.3–26.2) | 23.8 (21.6–26.5) | 26.3 (23.9–29.6) | 24.1 (21.7–26.7) | < 0.01 |
| Diabetes, n (%) ^b^ | 640 (17.4) | 262 (30.7) | 253 (64.1) | 335 (26.9) | < 0.01 |
| Child-Pugh class ^c^  A, n (%)  B, n (%)  C, n (%) | 2675 (72.5)   561 (15.2)  136 (3.7) | 606 (71)  174 (20.4)   32 (3.7) | 308 (77.8)  72 (18.2)  15 (3.8) | 839 (67.3)  219 (17.6)  52 (4.2) | < 0.01 |
| Maximal tumor size (cm) | 3.0 (2.0–6.1) | 3.4 (2.0–6.0) | 3.0 (2.0–5.2) | 4.3 (2.5–8.0) | < 0.01 |
| Number of nodules ^d^  Single, n (%)  2-3, n (%)  > 3, n (%) | 1867 (51.2)  1046 (28.6)  733 (20.2) | 446 (52.5)  208 (24.5)  200 (23.0) | 239 (60.4)  102 (25.7)  55 (13.9) | 656 (53.6)  267 (21.8)  325 (24.6) | < 0.01 |
| Vascular invasion, n (%) ^e^ | 446 (12.3) | 79 (9.4) | 36 (9.2) | 127 (10.3) | < 0.01 |
| Extrahepatic metastasis, n (%) ^f^ | 331 (9.1) | 65 (7.8) | 22 (5.6) | 165 (13.4) | < 0.01 |
| AFP (ng/mL) | 21.0 (5.0–292) | 9.0 (4.5–60) | 7.3 (4.0–51) | 14.1 (4.0–301) | < 0.01 |
| BCLC stage ^g^  0, n (%)  A, n (%)  B, n (%)  C, n (%)  D, n (%) | 487 (13.3)  1246 (34.1)  721 (19.7)  724 (19.8)  112 (3.1) | 87 (10.2)  342 (40)  205 (24)  166 (19.4)  26 (3.0) | 66 (16.7)  162 (40.9)  102 (25.8)  42 (10.6)  12 (3.0) | 82 (6.6)  458 (36.9)  231 (18.6)  299 (24.1)   66 (5.3) | < 0.01 |
| Initial treatment ^h^  Surgery, n (%)  Ablation, n (%)  Embolization, n (%)  Radiation, n (%)  Systemic therapy, n (%)  Hepatic arterial infusion, n (%)  Heavy Particle, n (%)  Transplantation, n (%)  No treatment, n (%) | 700 (18.9)  865 (23.4)  1091 (29.5)  56 (1.5)  347 (9.4)  94 (2.5)  7 (0.2)  43 (1.2)  491 (13.3) | 138 (16.2)  184 (21.5)  293 (34.3)  14 (1.6)  71 (8.3)  27 (3.2)  3 (0.4)  3 (0.4)  121 (14.2) | 66 (16.7)  109 (27.5)  138 (34.8)  8 (2.0)  18 (4.5)  8 (2.0)  2 (0.5)  9 (2.3)  38 (9.6) | 273 (21.9)  215 (17.2)  275 (22.0)  29 (2.3)  151 (12.1)  24 (1.9)  5 (0.4)  9 (0.7)  267 (21.4) | < 0.01 |
